# Supplementary material for: Modulation Effects of Toxoplasma gondii Histone H2A1 on Murine Macrophages and Encapsulation with Polymer as a Vaccine Candidate
Source: Vaccines (Basel). 2020 Dec 3;8(4):731. doi: 10.3390/vaccines8040731 (PMC7761694; doi:10.3390/vaccines8040731)
Supplement: Supplementary file 1 [file vaccines-08-00731-s001.pdf]

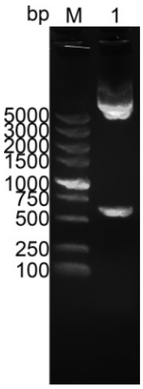

Figure S1. Double digestion analysis of the recombinant plasmid pET32a/H2A1. Lane M: DNA molecular weight marker DL5000; Line 1: plasmid pET32a/H2A1 digested by *Eco* RI and *Hind* III.

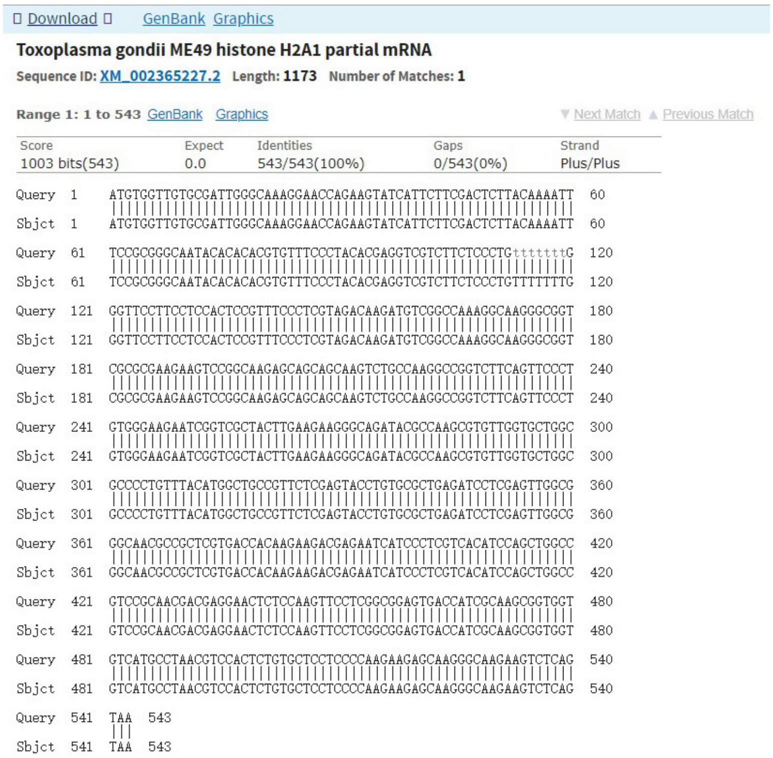

Figure S2. Sequence alignment results of the recombinant plasmid pET32a/H2A1. Compared with the nucleotide sequences of H2A1 gene (Genbank: XM_002365227), sequence analysis was conducted through the Blast program online (http://www.blast.ncbi.nlm.nih.gov/blast.cgi).

Table S1. Effects on proliferation at different concentrations (0, 5, 10, 20, 40, 80 μg/ml) of rTgH2A1 on the murine macrophages. Results were evaluated using one-way ANOVA analysis followed by Dunnett's test and shown as mean of the OD450 ± standard deviation.

Group	OD value	*P* value[a]	*P* value[b]
Blank	0.401 ± 0.016	-	0.9980
Control	0.408 ± 0.018	0.9980	-
5 μg/mL rTgH2A1	0.391 ± 0.015	0.9892	0.8892
10 μg/mL rTgH2A1	0.402 ± 0.017	> 0.9999	0.9982
20 μg/mL rTgH2A1	0.446 ± 0.019	0.1614	0.2857
40 μg/mL rTgH2A1	0.474 ± 0.030	0.0111	0.0218
80 μg/mL rTgH2A1	0.367 ± 0.017	0.3665	0.2134

[a] and [b] were compared with blank group and His tagged protein group, respectively.
